# CASC2c as an unfavorable prognosis factor interacts with miR-101 to mediate astrocytoma tumorigenesis

**DOI:** 10.1038/cddis.2017.11

**Published:** 2017-03-02

**Authors:** Changhong Liu, Yingnan Sun, Xiaoling She, Chaofeng Tu, Xiping Cheng, Lin Wang, Zhibin Yu, Peiyao Li, Qing Liu, Honghui Yang, Guiyuan Li, Minghua Wu

**Affiliations:** 1 Hunan Provincial Tumor Hospital and the Affiliated Tumor Hospital of Xiangya Medical School, Central South University, Changsha 410013, China; 2 Cancer Research Institute, School of Basic Medical Science, Central South University, Changsha 410078, China; 3 Key Laboratory of Carcinogenesis and Cancer Invasion, Ministry of Education, Changsha 410078, China; 4 Key Laboratory of Carcinogenesis, Ministry of Health, Changsha 410078, China; 5 Second Xiangya Hospital, Central South University, Changsha 410013, China; 6 Regeneron Pharmaceuticals, Tarrytown, NY 10591, USA; 7 Department of Neurosurgery, University of Michigan Medical School, Ann Arbor, MI 48109, USA; 8 Xiangya Hospital, Central South University, Changsha 410013, China; 9 Third Xiangya Hospital, Central South University, Changsha 410013, China

## Abstract

miR-101 has been suggested as a tumor suppressor, but the promoter methylation and loss of heterozygosity didn't contribute to its low expression in astrocytoma. We investigated the role of a new long non-coding RNA CASC2c binding with miR-101. High CASC2c was positively correlated with astrocytoma progression, and an unfavorable prognosis factor for patients. Knockdown CASC2c inhibited proliferation and tumorgenesis. Overexpression of CASC2c promotes the malignant characteristic of astrocytoma cells.CASC2c directly bound miR-101 and mediated pre-miR-101 processing into mature miR-101, and functions as a competitor of miR-101 target genes such as *CPEB1*. Patients who possessed both low CASC2c and high miR-101 had a longer survival than those of low CASC2c alone or high miR-101 alone. In summary, CASC2c plays the onco-RNA role in the tumorgenesis of astrocytoma by acting as a decoy miR-101 sponge. Combination of low expression of CASC2c and high expression of miR-101 has an important referential significance to evaluate the prognosis of patients.

miR-101 has been reported to show low expression and act as a tumor suppressor in various solid tumors. Researches indicated that the decreased expression of miR-101 is not due to the promoter hypermethylation, but the loss of heterozygosity is one of the reasons for decreased expression of miR-101 in bladder transitional cell carcinoma.^1^ Our previous data demonstrated that miR-101 is a tumor suppressor in astrocytoma,^2^ but they are not known about the decreased expression of miR-101 in astrocytoma. A growing volume of literature has demonstrated that long non-coding RNAs function as a nature decoy or a molecular sponge in modulating the biological functions of miRNAs.^3, 4, 5^ LncRNAs are reported as a biomarker for predicting survival and the diagnosis of multiple diseases.^6, 7, 8^ Recently, the function of lncRNAs have been widely recognized, including controlling gene transcription,^9^ regulating gene expression through modulation of chromatin remodeling,^10^ posttranscriptional mRNA processing^11^ and interacting with miRNA as a competing endogenous RNA (ceRNA) to participate in the expression regulation of target genes.^12, 13^

CASC2 (cancer susceptibility candidate 2; formerly C10orf5) has been isolated from chromosome 10q26 in endometrial cancer, and its alternative splicing of five exons of CASC2 generates three different mRNAs: CASC2a, CASC2b and CASC2c.^14^ CASC2a encodes a protein of 102 amino acids, but there is no similarity to known protein in the databases,^14^ and CASC2a transcript was found to be low expression in endometrial and colorectal cancer, whereas there is no alteration in other cancer type such as hepatocellular, renal and adrenal carcinomas.^15^ Up to date, CASC2a has been identified as a long non- coding RNA that suppressed malignancy in human glioma through miR-21.^16^ However, there is not known reports about the expression and functions of CASC2b and CASC2c.

In this study, we found that it is not sufficient enough for us to predict the CpG island in the promoter of pre-miR-101-1 and pre-miR-101-2 by online software; LOH in chromosome 1p31 (pre-miR-101-1) and 9p24 (pre-miR-101-2) did not contribute to the lower expression of miR-101 in astrocytoma. We verified that CASC2c is a long non-coding RNA. High expression of CASC2c was correlated with astrocytoma progression and an unfavorable prognosis marker. CASC2c and miR-101 repressed reciprocally and formed a RNA complex with targets of miR-101. They also mutually constrained and regulated each other in astrocytoma. Our data first provided new insights into the molecular function of CASC2c as well as its regulatory mechanisms.

## Results

### CASC2c is correlated with malignant progression of astrocytoma

**Figure 1:**
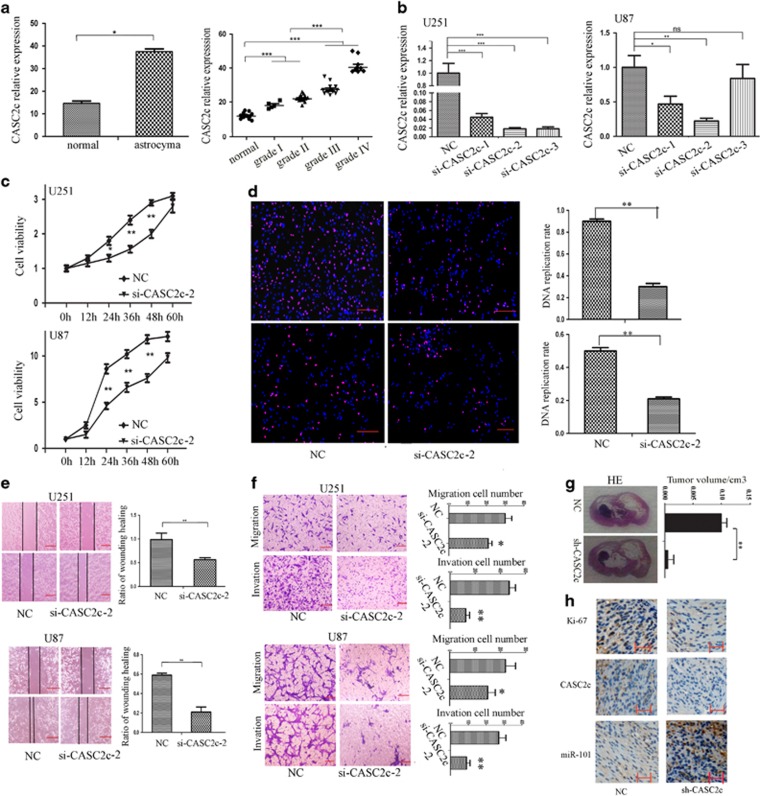
CASC2c is correlated with tumorigenicity and progression in astrocytoma. (**a**) Left, the expression of CASC2c in normal brain tissues and astrocytoma was determined by real-time qPCR. Data presented as mean±S.E.M. of three independent experiments; *P<0.05. Right, the level of CASC2c in different status of pathology classification was measured by real-time qPCR. Data presented as mean±S.E.M. of three independent experiments; ***P<0.001. (**b**) CASC2c expression level was evaluated using real-time qPCR in nc/si-CASC2c-transfected U251 and U87 cells. Data presented as mean±S.E.M. of three independent experiments; *P<0.05, **P<0.01, ***P<0.001. (**c**) CCK8 assay was performed to determine the viability of U251 and U87 cells that transfected nc/si-CASC2c-2.Data shown are the mean ±S.E.M. of three independent experiments; *P<0.05, **P<0.01. (**d**) EDU assay was applied to assess cell proliferation of U251 and U87 cells that transfected nc/si-CASC2c-2. Data shown are the mean±S.E.M. of three independent experiments; red scale bars, 200 *μ*m; **P<0.01. (**e**) Wound healing assay measured cell migration of U251 and U87 cells that transfected nc/si-CASC2c-2. Data shown are the mean±S.E.M. of three independent experiments; red scale bars, 200 *μ*m; **P<0.01. (**f**) Transwell assay and matrigel-coated transwell assay were performed in U251 and U87 cells that transfected si-CASC2c-2. Data shown are the mean±S.E.M. of three independent experiments; red scale bars, 50 *μ*m; *P<0.05, **P<0.01. (**g**) H&E staining of shCAS2c-induced tumors in the coronal section of rats. **P<0.01. (**h**) The expression of CASC2c and miR-101 in intracranial-transplanted tumors were detected by *in situ* hybridization, the expression of Ki-67 in intracranial-transplanted tumors were detected by immunohistochemical staining. Scale bars, 200 *μ*m

CpG island in the promoter of pre-miR-101-1 and pre-miR-101-2 has not been founded by online software (Supplementary Figure S1); LOH in chromosome 1p31(pre-miR-101-1) and 9p24 (pre-miR-101-2) did not contribute to the lower expression of miR-101 in astrocytoma (Supplementary Figure S1). So we performed a search for lncRNAs that have complementary base pairing with miR-101 by software program DIANA LAB and miRanda (Memorial Sloan-Kettering Cancer Center, New York City, USA). There were 18 lncRNAs predicted to form putative binding sites with miR-101 (Supplementary Table S2), and only 6 lncRNAs have been confirmed to show significantly different expression between astrocytoma and normal brain tissues (Supplementary Figure S2). CASC2c increased significantly in astrocytoma tissues (Figure 1a left), and the increased expression levels of CASC2c were positively correlated with the status of malignant progression of astrocytomas (Figure 1a right).

We further analyzed the CASC2 gene family and found that CASC2 has five exons named I,II,III, IV (IVa, IVb, IVc) and Va that can generate three transcripts: CASC2a, the length is 3285 bp including I, II, III, IVa, Va, and has been authenticated a lncRNA,^14, 15, 16^ CASC2b, the length is 1658 bp including I, II, III, IVb and CASC2c, its length is 3221 bp including I, II, III, IVc (Supplementary Figure S3). Although CASC2c encodes ~80 amino acids through ORF finder (Supplementary Figure S3), we could not find significant homology for the ~80 amino acids of the predicted protein with any known protein. We also didn't find protein domains that have confirmed the existence by using SMART and Coding Potential Calculator (Supplementary Figure S3). As shown in Supplementary Figure S4, CASC2a expression levels were significantly decreased in glioma. So we think that CASC2c acts as an lncRNA, but it functions completely differently from CASC2a in glioma.^16^ CASC2c overexpression promotes the malignant characteristic of astrocytoma cells (Supplementary Figure S5).

### Knockdown of CASC2c suppresses tumorigenicity of astrocytoma cells

To further detect its biological role in astrocytoma, we designed three CASC2c siRNA (si-CASC2c-1, si-CASC2c-2 and si-CASC2c-3) to determine the interference effect because CASC2c is highly expressed in U251 and U87 cells (Supplementary Figure S6). Compared with the si-NC, si-CASC2c-2 has the best interference effect (Figure 1b). Thus, si-CASC2c-2 was used for the further study. Simultaneously, we found that the expression of CASC2a and CASC2c is decreased by knockdown CASC2c (Supplementary Figure S7).

Subsequently, knockdown CASC2c resulted in a significant decrease in viability using CCK8 assay (Figure 1c) and EDU assays (Figure 1d). Cell cycle analysis showed that knockdown CASC2c increased the number of U251 and U87 cells in G0/G1 phase (Supplementary Figure S8). Wound healing assay and migration assay showed that the migration of U251 and U87 cells was significantly inhibited by knockdown CASC2c (Figures 1e and f). And as shown in Figure 1f, the invasion of U251 and U87 was inhibited in the si-CASC2c-2 groups.

We further investigated the tumorigenicity effect of knockdown CASC2c on the Sprague–Dawley rats intracranial orthotopic-transplanted astrocytoma. We constructed pSuper-CASC2c-shRNA plasmid, and stably expressed in U251 cells (Supplementary Figure S9). U251 cells with or without CASC2c shRNA were intracranially transplanted into the rat brain parenchyma. After 30 days, HE staining revealed that CASC2c shRNA was accompanied by significantly reduced growth of the intracranial transplanted tumors (Figure 1g). ISH and IHC demonstrated that both expressions of CASC2c and Ki-67 decreased in shRNA-CASC2c-xenografted tumors, and expressions of miR-101 were increased (Figure 1h). The above results manifested that knockdown CASC2c had tumor-suppressive effects on astrocytoma cells.

### *CASC2c* is a target gene of miR-101 and regulated directly by miR-101

**Figure 2:**
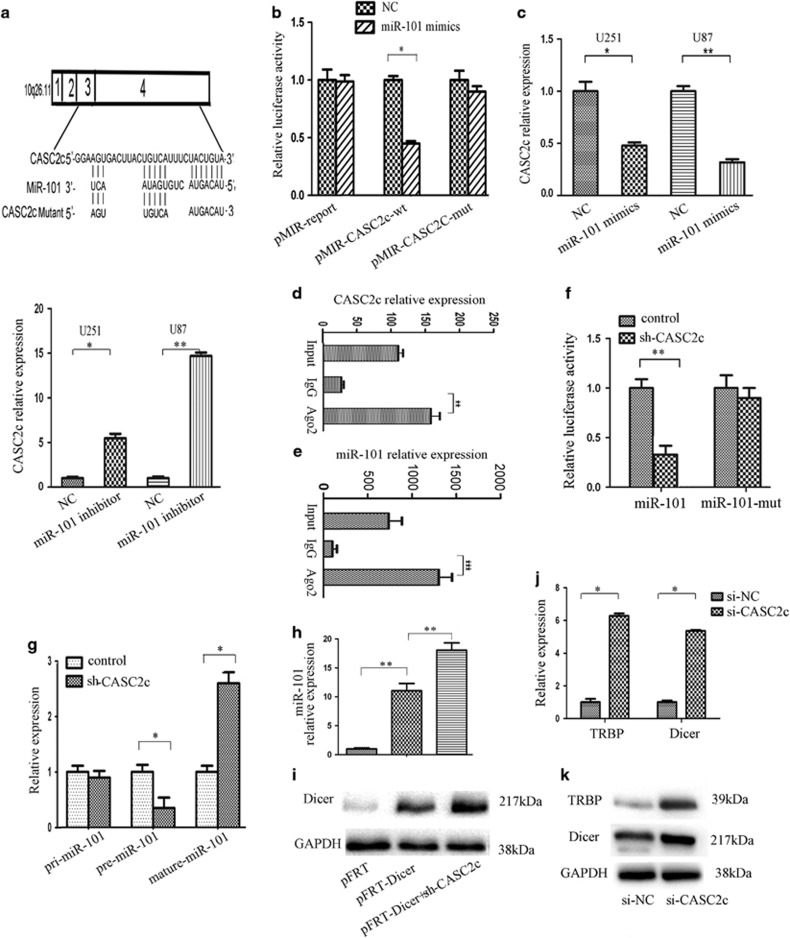
CASC2c and miR-101 existed in RISC complex simultaneously and formed a reciprocal repression loop between miR-101 and CASC2c. (**a**) Alignment of potential CASC2c base pairing with miR-101 was identified by using DIANA LAB (http://diana.cslab.ece.ntua.gr/microT/). (**b**) Relative fluorescence activity in U251 cells co-transfected with pMIR-REPORT-WT/mutant CASC2c and miR-101 or the negative control. Data presented as mean±S.E.M. of three independent experiments; *P<0.05. (**c**) Expression of CASC2c was assayed by real-time qPCR in miR-101 mimics/miR-101 inhibitor-transfected U251 and U87 cells. Data presented as mean±S.E.M. of three independent experiments; *P<0.05, **P<0.01. (**d** and **e**) Association of CASC2c and miR-101 with Ago2 in U251 cells. CASC2c and miR-101 expression levels were detected using real-time qPCR. Data presented as mean±S.E.M. of three independent experiments; **P<0.01, ***P<0.001. (**f**) Relative fluorescence activity in U251 cells co-transfected with the pMIR-miR-101 wild-type reporter plasmid (or the corresponding pMIR-miR-101mutant reporter) and shCASC2c/pcDNA3.1-CASC2c plasmid or the negative control. Data presented as mean±S.E.M. of three independent experiments; **P<0.01. (**g**) The expression of pri-miR-101, pre-miR-101-1 and mature miR-101 was measured by real-time qPCR in NC- or shCASC2c-transfected U251 cells. Data presented as mean±S.E.M. of three independent experiments; *P<0.05. (**h**) The expression of miR-101 was analyzed through real-time qPCR in pFRT plasmid, pFRT-Dicer or pFRT-Dicer-transfected U251 cells. Data presented as mean±S.E.M. of three independent experiments; *P<0.05, **P<0.01. (**i**) The expression of Dicer was measured by western blot in pFRT plasmid, pFRT-Dicer or pFRT-Dicer-transfected U251 cells. (**j**) The expression of Dicer and TRBP was evaluated using real-time qPCR in control/si-CASC2c-transfected U251 cells. Data presented as mean±S.E.M. of three independent experiments; *P<0.05. (**k**) The expression of Dicer and TRBP was evaluated using western blot in control/si-CASC2c-transfected U251 cells

Bioinformatics analysis for miR-101 recognition sequences on CASC2c revealed the presence of three highly conserved miR-101 sites listed in Supplementary Figure S10, the CASC2c cDNA (RLuc-CASC2c-wt) and mutant derivatives lacking the putative miR-101 recognition sequences (RLuc-CASC2c-Δ1, RLuc-CASC2c-Δ2 and RLuc-CASC2c-Δ3) were cloned downstream of the luciferase gene and transfected in U251 cells with miR-101 nc or miR-101 mimics. Supplementary Figure S11 showed that luciferase expression was reduced by 70% respect to the control when RLuc-CASC2c-Δ1 and RLuc-CASC2c-Δ2 were respectively expressed. On the contrary, when the RLuc-CASC2c-Δ3 plasmid was used, the luciferase expression repression was restored (Supplementary Figure S11).These data demonstrated that CASC2c binds miR-101 through the third binding site. To verify the direct binding between CASC2c and miR-101 by the third binding site, we sub-cloned the third recognition sequence of CASC2c including the predicted miR-101 recognition site (wild type) or the mutated sequence (mutant type) into pMIR-reporter plasmids (Figure 2a). miR-101 reduced the luciferase activity of wild-type CASC2c reporter vector. Luciferase activity was hardly comparable to that of control cells when U251 cells co-transfected with CASC2c-mutant reporter vector and miR-101 mimics (Figure 2b). Subsequently, real-time qPCR indicated that miR-101 overexpression significantly decreased the expression of CASC2c, whereas miR-101 knockdown increased the expression of CASC2c (Figure 2c).

It is well known that miRNAs exert their gene silencing functions through RNA-induced silencing complex.^23^ As the core component of RISC was Ago2, RIP was performed on U251 cell extracts using antibodies against Ago2. Both CASC2c and miR-101 in U251 cells were enriched in Ago2 immunoprecipitates relative to control IgG immunoprecipitates (Figures 2d and e). As shown in Supplementary Figure S12, knockdown CASC2c resulted in higher enrichment of miR-101 in Ago2 complex. These results indicate that CASC2c and miR-101 are in RISC complex. And there exists a direct binding between CASC2c and miR-101.

### Knockdown of CASC2c negatively regulates the expression of miR-101

We have confirmed that CASC2c is regulated directly by miR-101. Whether CASC2c also regulates the expression of miR-101, and if they are a reciprocal repression loop (Supplementary Figure S13). We observed that CASC2c decreased the luciferase activity of wild-type miR-101 reporter vector, whereas shCASC2c increased the luciferase activity of wild-type miR-101 reporter vector (Figure 2f). Luciferase activity didn't increase when we co-transfected miR-101-mut reporter plasmid and shCASC2c plasmid in U251 cells (Figure 2f).

These data confirmed that *miR-101* is the target gene of CASC2c. Then we detected the expression of miR-101 after knockdown or overexpression CASC2c (Supplementary Figure S14). The expression of mature miR-101 was increased in U251 cells transfected with CASC2c shRNA plasmid (Figure 2g). We further found that knockdown CASC2c decreased the expression of pre-miR-101, and had no effect on the expression of pri-miR-101. These data prompted the idea that negative regulation of CASC2c on mature miR-101 might be through a Dicer processing mechanism.

To further explore negative regulation of knockdown CASC2c on mature miRNA-101 through impacting the processing of Dicer. Ectopic expression of Dicer significantly upregulated the level of miR-101 in U251 cells (Figure 2h), whereas, knockdown CASC2c promoted the increase of Dicer (Figure 2i), and increased Dicer-induced mature miR-101 expression (Figure 2h). TRBP is known to be important for Dicer binding to its siRNA product, it has also shown to stabilize Dicer.^24, 25^ Knockdown CASC2c substantially increased Dicer and TRBP expression in U251 cells (Figures 2j and k). CASC2c negative regulated the expression of mature miR-101 by affecting Dicer processing from pre-miR-101 to mature miR-101.

Having confirmed that CASC2c was a target of miR-101, the role of CASC2c in miR-101-induced inhibition on astrocytoma cells remains unclear. Supplementary Figure S15 showed that CASC2c in U251 and U87 cells knockdown largely reversed the promotion effect of miR-101 inhibitor on cell proliferation, migration and invasion. These results strongly suggested that CASC2c played a crucial role in miR-101-induced inhibitory effects on astrocytoma cells.

### CASC2c regulates target genes of miR-101

**Figure 3:**
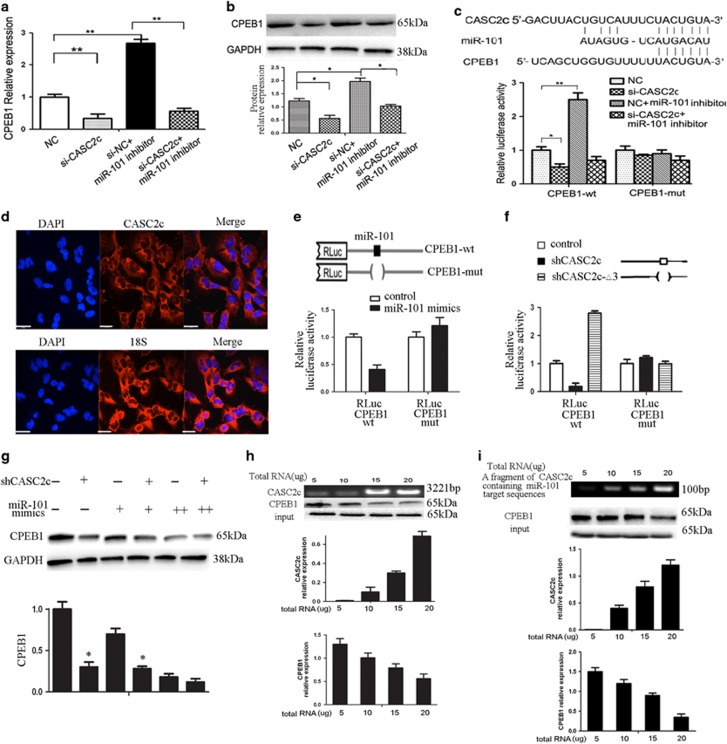
CASC2c competes to combine miR-101 by repelling CPEB1. (**a** or **b**) The expression of miR-101 was analyzed through real-time qPCR (**a**) and western blot (**b**) in si-CASC2c, miRNA-101 inhibitor or miRNA-101 inhibitor-transfected U251 cells. Data presented as mean±S.E.M. of three independent experiments; *P<0.05, **P<0.01. (**c**) Upper, nucleotide resolution of miRNA-binding sites in CASC2c and CPEB1. Below, relative fluorescence activity in U251 cells co-transfected with pMIR-REPORT-WT/mutant 3′-UTR CPEB1 and miR-101 inhibitor or negative control, pMIR-REPORT-WT/mutant 3′-UTR CPEB1, si-CASC2c and miR-101. Data presented as mean±S.E.M. of three independent experiments; *P<0.05, **P<0.01. (**d**) RNA fluorescence *in situ* hybridization showing the localization of CASC2c, the nucleus is counterstained with DAPI. Scale bar, 29*μ*m. (**e**) Relative fluorescence activity in U251 cells co-transfected with RLuc-CPEB1-wt or RLuc-CPEB1-mut and miR-101 ncor miR-101 mimics. Data presented as mean ±S.E.M. of three independent experiments; *P<0.05, **P<0.01. (**f**) Relative fluorescence activity in U251 cells co-transfected with RLuc-CPEB1-wt or RLuc-CPEB1-mut and shCASC2c or shCASC2c-△3 plasmid. Data presented as mean±S.E.M. of three independent experiments; *P<0.05, **P<0.01. (**g**) The expression of CPEB1 was analyzed through western blot in U251 cells transfected with different combinations of shCASC2c and miR-101 mimics. (+) corresponds to 1.5 mg shCASC2c and to 40 ng of miR-101 mimics, whereas (++) corresponds to 200 ng of miR-101 mimics. Data presented as mean±S.E.M. of three independent experiments; *P<0.05. (**h**) The expression of CASC2c and CPEB1 was detected by real-time qPCR and western blot in U251 cells after adding variant amount of CASC2c into miR-101-CPEB1 reaction mixture. Data presented as mean±S.E.M. of three independent experiments. (**i**) The expression of CASC2c and CPEB1 was detected by real-time qPCR and western blot in U251 cells after adding variant amount of the fragment of CASC2c that containing miR-101 target sequences into miR-101-CPEB1 reaction mixture. Data presented as mean±S.E.M. of three independent experiments

We next investigated the effect of CASC2c on miR-101 target genes. We have verified that *CPEB1* is a target gene of miR-101.^2^ We found knockdown CASC2c inhibited the expression of CPEB1, whereas co-transfection of miR-101 inhibitor attenuated this inhibition (Figures 3a and b).

Several studies have shown a link between lncRNA and miRNA target genes by means of the same miRNA-responsive element,^26^ so we guess this phenomenon exists between CASC2c and CPEB1 because they displayed the same miR-101-dependent regulation pattern. As illustrated in Figure 3c, CASC2c knockdown decreased the luciferase activity in the Luc-CPEB1-3'-UTR- transfected U251 cells, which was rescued by miR-101 inhibitor.

### CASC2c competes to combine miR-101 by repelling CPEB1

Analysis of CASC2c by RNA fluorescence *in situ* hybridization revealed CASC2c is localized in cytoplasm (Figure 3d). We observed significant enrichment of CASC2c in the cytoplasm by real-time qPCR (Supplementary Figure S16). Next, we determined the ability of CASC2c to decoy the dual targeting miR-101 from luciferase reporters. The 3′UTR of CPEB1 was fused to the luciferase-coding region (RLuc-CPEB1-wt) and transfected in U251 cells with miR-101 mimics in parallel to miR-101 nc. Luciferase assays showed that CPEB1 was one of the targets of miR-101 (Figure 3e). The use of RLuc-CPEB1-mut in the miR-101 recognition sites confirmed the specificity of the repressing activity.

RLuc-CPEB1-wt constructs were subsequently transfected in U251 cells with shCASC2c or mutant derivatives (shCASC2c-Δ3) (Figure 3f). Luciferase assays indicated that, in the presence of the shCASC2c, both 3′UTR reporter constructs were downregulated (Figure 3f). These indicated that CASC2c, by binding miR-101, acts as a decoy abolishing miR-101-repressing activity on CPEB1 3′ UTR. These effects were lost when RLuc-CPEB1-mut was utilized. This proves that there is a specific crosstalk between the CASC2c and CPEB1 through competition for miR-101 binding.

If CASC2c effectively acts as a decoy, one would expect that the relative concentration of the decoy and the miR-101 affects the expression of the target CPEB1. We gradually increased the amount of miR-101 in the presence of decreasing amount of shCASC2c. As shown Figure 3g, endogenous CPEB1 was low in excess of shCASC2c and are gradually reduced when miR-101 are increased. The data further proved that there was an interaction among the three components.

Next, we incubated miR-101 with purified CPEB1 protein to form a protein: RNA complex, and added CASC2c to the reaction mixture. Following this route, real-time qPCR and western blot were used to determine the residual amount of CASC2c and CPEB1. After adding the variant amount of RNA, we found that CASC2c significantly competed to combine miR-101 (Figure 3h). In addition, we constructed a fragment of CASC2c that containing miR-101 target sequences, then added the fragment to miR-101–CPEB1 reaction mixture, and performed real-time qPCR and western blot to detect the expression of CASC2c and CPEB1. The results showed that CASC2c significantly competed to combine miR-101 after adding variant amount of the fragment of CASC2c that containing miR-101 target sequences (Figure 3i).

### Patients with astrocytoma expressing high CASC2c levels have a poor clinical outcome

**Figure 4:**
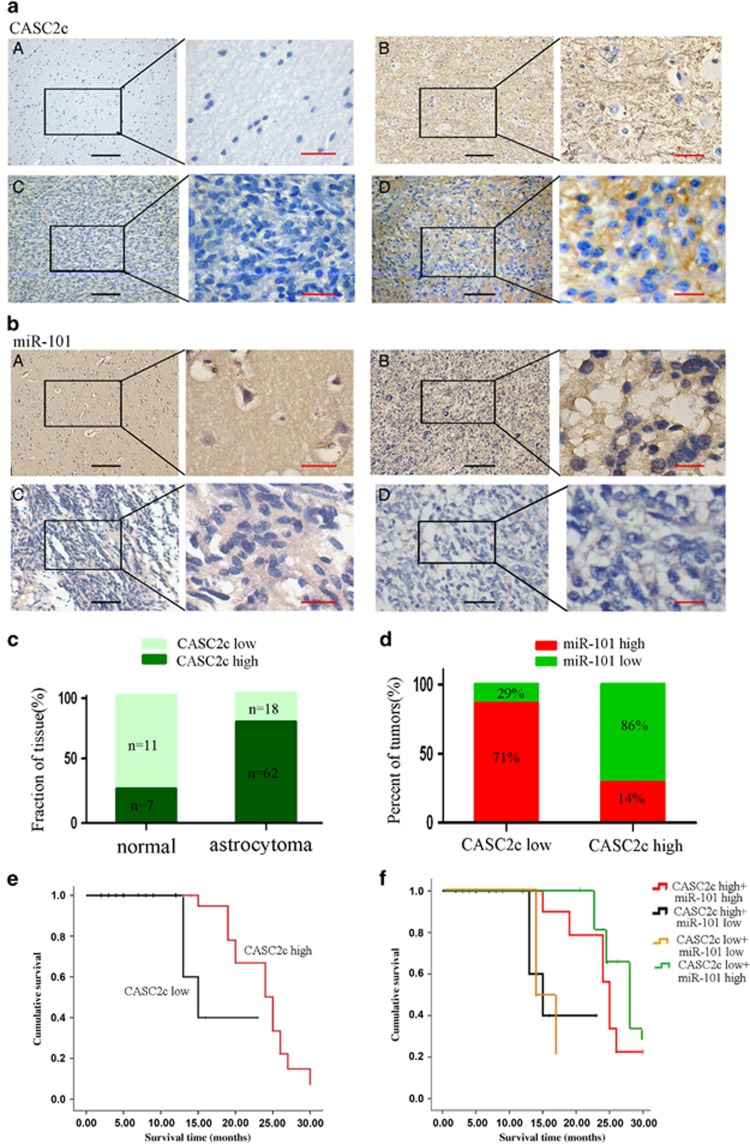
Association between miR-101 and CASC2c expression in astrocytoma clinical samples. (**a** and **b**) The expression level of CASC2c and miR-101 was detected by *in situ* hybridization. Black scale bars, 200 *μ*m; red scale bars, 20 *μ*m. (**c**) ISH score breakdown for a panel of normal tissues and astrocytoma. Each patient sample was scored from triplicate representative tumor cores, and the average CASC2c ISH score was recorded as low (score⩽8) or high (score>8). (**d**) The correlation between CASC2c expression and miR-101 levels were analyzed using Spearman's rank test. (**e**) Kaplan–Meier analysis for overall survival in 80 astrocytomas in high- and low-risk groups based on CASC2c expression levels. (**f**) Kaplan–Meier analysis for overall survival in 80 astrocytomas in high- and low-risk groups based on CASC2c and miR-101 expression levels

To confirm CASC2c expression may provide a clinical opportunity to identify tumors, we developed CASC2c and miR-101 ISH by using 80 paraffin-embedded astrocytoma tissue samples and 18 normal brain tissues (Figures 4a and b), examined CASC2c expression directly in astrocytoma tissue samples (Figure 4c). Interestingly, CASC2c was associated with miR-101 expression but was inversely correlated with miR-101 expression (Supplementary Table S3 and Figure 4d). CASC2c expression was significantly higher, whereas miR-101 expression was lower in astrocytoma tissues. In CASC2c low-expression group, the percentage of miR-101 high expression accounted for 71%, and low expression shared 29%. However, in CASC2c high-expression group, the percentage of miR-101 high expression accounted for 14%, and low expression shared 86%. Spearman's correlation analysis showed a negative relationship between CASC2c and miR-101 expression level (Figures 4d; *r*=−0.0657).

Our previous study has reported astrocytoma patients who had lost miR-101 expression were accompanied by a markedly reduced overall survival time.^2^ Here, we analyzed the relationship between clinic-pathological characteristics and CASC2c expression levels in patients with astrocytoma. As shown in Table 1, the higher expression of CASC2c also markedly correlated with clinical stage and death of patients, but it is not correlated with gender and age.

To further examine the effect of expression of CASC2c on the survival of astrocytoma patients, we employed the Kaplan–Meier analysis to plot the survival curve of 80 astrocytoma patients. Figure 4e illustrated the Kaplan–Meier survival plots for astrocytoma patients with different expression levels of CASC2c. Univariate survival analysis showed that the overall survival rate was significantly lower in CASC2c high expression (ISH score>8) patients compared with that in CASC2c low-expression patients (ISH score⩽8) (*P*=0.008). Furthermore, we examined the prognostic value of CASC2c and miR-101 on different subgroups of astrocytoma patients. Patients with low CASC2c and high miR-101 expression had longer survival than other subgroups (Figure 4f).

To determine whether CASC2c was the independent prognostic parameters for astrocytoma, a multivariate Cox proportional hazard regression analysis was carried out to further evaluate the expression of CASC2c as the prognostic factors. As summarized in Table 2, the multivariate analysis proved that high expression of CASC2c in astrocytoma was independent prognostic factor of overall survival.

## Discussion

In the present study, we first confirmed that decrease of miR-101 is not due to the promoter hypermethylation and loss of heterozygosity in astrocytoma. The higher expression of CASC2c is one of reasons for miR-101 low expression. We revealed that CASC2c is a long non-coding RNA, and provided evidence that high expression of CASC2c was in astrocytoma. Knockdown CASC2c suppressed the proliferation, migration and invasion *in vitro* and astrocytoma tumorgenesis *in vivo*. And overexpression of CASC2c promoted the malignant characteristic of astrocytoma cells. As an important nucleotide molecule, lncRNAs have always shown multiple roles in different organisms.^26^ Although both CASC2a and CASC2c belong to CASC2 gene family, genomic and cDNA sequence comparisons revealed that they share the first three exons but contain different downstream exons. CASC2a was expressed in a lower level in glioma tissues. Therefore, as CASC2a has tumor suppressor role in melanoma,^15^ colon cancer^15^ and glioma,^16^ our study is the first to confirm that CASC2c plays completely different role from CASC2a. CASC2c acted as an onco-RNA to be involved in the tumorgenesis and progression of astrocytoma.

Emerging evidence suggests lncRNAs communicate with ncRNAs, mRNAs, proteins and genomic DNA, and act as tethers, guides, decoys and scaffolds.^12, 27, 28^ LncRNAs can participate in ceRNAs regulatory network and act as endogenous miRNA sponges to compete for binding of miRNA through MRE, which is 'the letters' of the RNA code.^29, 30^ ceRNAs and miRNAs repress reciprocally and form a double-negative feedback loop.^31, 32^LncRNA MD1 binding with miR-133 and miR-135, acts as ceRNA for their mRNA targets, including MAML1 and MEF2C.^12^ In the current study, DIANA LAB and miRanda software were used to predict 18 lncRNAs, which bind with the miR-101, including CASC2c. We demonstrated CASC2c bound to miR-101 directly by MRE of miR-101, and there was reciprocal repression between CASC2c and miR-101. We also first found that high level of CASC2c positively regulated the expression of pre-miR-101, but in the processing from pre-miR-101 to mature miR-101, CASC2c negatively regulated the expression of Dicer, and inhibited the expression of mature miR-101.

Conversely, CASC2c is also the target of miR-101, and commonly exists in the RISC complex with miR-101. It has been reported that miR-101 are involved in a series of cellular activities,^33, 34, 35^ and there are potential interactions between miR-101 and transcription factors in normal and pathological states of cells. miR-101 could participate in the development of cervical cancer and is partly through loss of inhibition of target gene *COX-2*.^36, 37^ miR-101 targets MYCN and inhibit the proliferation and clonogenic growth of MYCN-amplified neuroblastoma cells.^38^ And miR-101 increases HIF1*α* protein levels by repressing VHL in normoxia condition.^39^ In addition, miR-101 regulates macrophage innate immune responses to LPS via targeting DUSP1.^40^ Our previous studies indicate that miR-101 not only directly regulates transcription factors expression by binding to 3′-UTR of genes, but also reverses its hypomethylation level to decrease gene expression through histone and DNA methylation modification, such as CPEB1, LMO3 and PRDM16.^2, 17, 18^ In this study, we found that CASC2c is localized in cytoplasm and regulated directly by miR-101.

At the same time, CASC2c functions as miR-101 sponge or as competitors of target gene *CPEB1* of miR-101 to compete for binding miR-101, and prevents reduction of CPEB1 by suppressing miR-101. CPEB1 is a dual-function protein. Our previous studies showed that the expression of CPEB1 was increased in glioma tissues compared with normal brain tissues.^2^

In light of these notions, we proved that depletion of CASC2c led to repression of CPEB1, notably, in conditions of CASC2c insufficient, titrated repression of CPEB1 could be obtained by increasing miR-101 levels. The data indicated a direct competition for miR-101 binding between CASC2c and CPEB1. Therefore, we assumed that in normal tissue, CASC2c, miR-101 and CPEB1 keep a balance and form a regulatory feedback loop consisting of mutual competitiveness restriction. However, in the astrocytoma, because of the abnormally high expression of CASC2c, or aberrant low expression of miR-101 or the overexpression of CPEB1 as a result of hypomethylation status of its promoter, the balance of this regulatory complex of CASC2c, miR-101 and CPEB1 was broken. Thinking about the complexity of molecular mechanism in tumor, we are unable to define a cause-and-effect relationship among CASC2c, miR-101 and CPEB1 for dysfunction. But the current study suggests that miR-101 may be a core unit, and MRE of miR-101 is an important functional sequence for the crosstalk among CASC2c, miR-101 and its target gene *CPEB1*. Low expression of miR-101 is one of the reasons for high expression of CPEB1, whereas the high expression of CASC2c may account for miR-101 low expression and overexpression of CPEB1, whereas ectopic miR-101 expression mediated tumor suppression of shCASC2c in astrocytoma.

Finally, we confirmed the prognostic value of CASC2c by using clinical specimens. We observed that the high expression of CASC2c was an unfavorable prognosis marker for astrocytoma patients. Patients with high expression of CASC2c had worse survival time than those with low expression of CASC2c. Moreover, patients with high levels of CASC2c tended to accompany with low levels of miR-101. On the contrary, patients with low levels of CASC2c tended to have high levels of miR-101. Patients who combined both low CASC2c expression and high miR-101 expression had a longer survival time than those with low CASC2c alone or high miR-101 alone. Therefore, we suggested that combination of CASC2c and miR-101 has an important referential significance to evaluate the prognosis in astrocytoma patients. To summarize, this finding may provide a new clue for the understanding of astrocytoma and CASC2c may represent a novel approach for the treatment and prognosis of astrocytoma patients.

## Materials and methods

### Human tissue samples and cells culture

Astrocytoma tissues and normal brain tissues were obtained from the Department of Neurosurgery, Xiangya Hospital, Hunan, China. Informed consent was obtained from all patients and the study was approved by the Ethics Committee. U251 and U87 cells were obtained from the Cell Center of Peking Union Medical College (Beijing, China), U251 cells were authenticated that it origins from ATCC by short tandem repeat profiling, U87 cells were authenticated that 93% is similar with glioblastoma cells from ATCC by short tandem repeat profiling and 97% is similar with glioblastoma cells from DSMZ by short tandem repeat profiling. And several of articles were published in journals by using U87 cell line including 'Cell Death and Disease'.^2, 17, 18^ HEB cell was a primary human normal glial cell and obtained from the Cell Center of Sun Yat-sen University (Guangzhou, China). We did not find HEB similar with human normal glial cell from ATCC by short tandem repeat profiling because this primary cell has not been recorded in ATCC. And several of articles were published in journals by using this cell line that from Cell Center of Sun Yat-sen University.^19, 20^ U251, U87 and HEB cells were cultured in 1640 containing 10% FBS and 1% antibiotic-antimycotic solution (Gibco, Grand Island, NY, USA).

### MicroRNAs, siRNAs, DNA plasmids and transfection

Cells transfection was performed using Lipofectamine 3000 (Invitrogen-Life Technologies, Carlsbad, CA, USA) as per the manufacturer's instructions.

### Real-time qPCR

This procedure was carried out as previously described.^2^ The primers were summarized in Supplementary Table S1.

### Western blotting

Western blot analysis was performed as previously described.^2^ The first antibodies were detected with goat polyclonal antibody for CPEB1 (Santa Cruz Biotechnology, Santa Cruz County, CA, USA), mouse monoclonal antibody for Ago2 (Abcam, Cambridge, UK), mouse monoclonal antibody for Dicer and GAPDH (Millipore, Danvers, MA, USA).The intensity of protein fragments was quantified using Image Lab software (Bio-Rad, Berkeley, CA, USA).

### Cell viability and EdU assays

Cell viability was evaluated using CCK8 (Beyotime Biotechnology, Suzhou, China). Cells (2 × 10^3^) were seeded in 96-well plates by different treatment, and cultured for different time points. CCK8 solution (20 *μ*l) was added to each well, and the plates were incubated at 37°C for 4 h. Absorbance was measured at 450 nm on a microplate reader (Bioteck, Arcugnano ,Vicenza, Italy). Proliferating cell count was measured using the Cell-Light EdU DNA Cell Proliferation Kit (RIBOBio Co., Guangzhou, China). The cells were seeded in 96-well culture plates and exposed to media with or without plumbagin 2 × 10^3^ cells per well were treated with 50 *μ*mol/l of EdU for 4 h at 37°C. After being fixed with 4% paraformaldehyde for 30 min, the cells were baptized with 0.5% Triton X-100 for 30 min and rinsed with PBS three times. Thereafter, the cells were exposed to 100 *μ*l per well of 1 × Apollo reaction cocktail for 30 min and incubated with 5 *μ*g/ml of DAPI to stain the cell nuclei for 15 min. Images were captured using a fluorescent microscope (Olympus, Tokyo, Japan). Experiments were repeated at least three times.

### Wound healing assay

Cells were cultured until they reached 90% confluence in 6-well plates. Cell layers were scratched using a 10 *μ* l tip to form wounded gaps, washed with PBS twice and cultured. We treated cells by hydroxycarbamide (10 *μ*g/ml). The wounded gaps were photographed at different time points and analyzed by measuring the distance of migrating cells from five different areas for each wound.

### Cell migration and invasion assays

Cell migration and invasion assays were performed using 24-well transwell chambers with 8 *μ*m pore size polycarbonate membrane (Corning Incorporated, Corning, NY, USA). Cells were seeded on the top side of the membrane (without Matrigel for cell migration assay) or seeded on the top side of the membrane pre-coated with Matrigel (BD, Franklin Lakes, NJ, USA) (for cell invasion ability assay). After 48 h of incubation, cells inside the upper chamber were removed with cottons swabs. Migrated and invaded cells on the lower membrane surface were fixed and then stained with 10% crystal violet. Five randomly fields were counted randomly in each well.

### Intracranial transplanted tumor model in rats

All experiments were carried out with the approval of the Animal Care and Use Committee of Central South University. U251 cells were cultured in 1640 medium in 6-well plates until they reached by 85–90% confluence, after which the medium was replaced with serum-free 1640. Four microgram of pSuper-CASC2c-shRNA plasmid was transfected in U251 cells by Lipofectamine 3000. After 12 h, the serum-free 1640 was replaced with 1640 containing 2% FBS. Approximately 48 h after the transfection, cells were digested with 0.05% trypsin and the cell suspensions were plated onto 50 ml culture flasks. The cells were cultured for 25–48 days in a medium containing 400 *μ*g/ml of G418 to select the transfectants. Stably transfected cells were identified by measuring the expression of CASC2c. Sprague–Dawley rats with 6 weeks old and 200–250 g were anesthetized by intraperitoneal injection of ketamine (40 mg/kg), and U251 cells were washed once with PBS and subcutaneously injected into brain parenchyma as previously described at a concentration of 1 × 10^6^ cells per mouse.^21^ The cyclophosphamide was injected (800 mg/kg) every 4 days.

### Luciferase reporter assay

This procedure was carried out as previously described.^22^ Firefly and Renilla reniformis luciferase activities were measured at 24 h after transfection by the dual luciferase reporter assay system (Promega, Madison, WI, USA).

### RNA-binding protein immunoprecipitation

About 2 *μ*g cells extract was mixed with agarose beads, which has already precipitation with protein antibody. Beads were washed briefly three times with GLB^+^ lysis and the retrieved protein was detected by western blot. The co-precipitated RNAs were detected by real-time qPCR. Total RNAs and controls were also assayed to demonstrate that the detected signals were from RNAs specifically binding to protein.

### *In situ* hybridization

miR-101 miRCURYTM LNA custom detection probes (Exiqon, Vedbaek, Denmark) and CASC2c custom detection probes (Boster, Wuhan, China) were used for ISH. Hybridization, washing and scanning were carried out according to the manufacturer's instructions. Image analysis and total gray value estimation were conducted by the GSM-2000P pathology image analysis system (Heima, Zhuhai, China).

### Statistical analysis

All experiments were analyzed with GraphPad Prism 5 (La Jolla, CA, USA). Differences between the different groups were tested using the Student's *t*-test or one-way ANOVA. The correlation between CASC2c expression and miR-101 levels was analyzed using Spearman's rank test. The relationships between the CASC2c expression and clinic-pathological parameters were examined using the *χ*2-test. The expression of CASC2c and patients' survival time was analyzed by single factor and multiplicity factors analysis, and OS curves were calculated using the Kaplan–Meier method by SPSS 15.0 program (SPSS Inc., Chicago, IL, USA). Data are expressed as means±S.E.M. from at least three independent experiments. A probability value *P*<0.05 was considered statistically significant.

## Figures and Tables

**Table 1:** Correlation between the clinicopathological factors and expression of CASC2c in astrocytoma

	CASC2c			
Variable		Low	High	*P*-value
Total (*N*=80)	*N*	38	42	
*Sex*				
Male	26	14(36.8%)	12(28.5%)	
Female	54	24(63.2%)	30(71.5%)	0.423
				
*Age*				
⩽42	43	22(57.9%)	21(50%)	
>42	37	16(42.1%)	21(50%)	0.555
				
*Grade*				
I+II	42	37(97.3%)	5(11.9%)	
III+IV	38	1(2.7%)	37(88.1%)	0.000
				
*Death*				
Yes	51	17(44.7%)	34(80.9%)	
No	29	21(55.3%)	8(19.1%)	0.001
				
*Chemoradiotherapy*				
Yes	51	21(55.3%)	30(71.4%)	
No	29	17(44.7%)	12(28.6%)	0.021

**Table 2:** Summary of multivariate analysis of Cox proportional hazards model for survival of patients with astrocytoma

Parameter		Multivariate	
	Hazard ratio	95% CI	*P*-value
*Gender*			
Female *versus* male	2.12	0.89–2.89	0.95
			
*Age*			
⩽42 *versus* >42	1.05	0.05–1.37	0.47
			
*Grade*			
I+II*versus* III+IV	0.98	0.68–1.15	0.06
			
*Treatment strategy*			
Combination RT-CT *versus* RT or CT	1.07	0.92–1.10	0.08
			
*CASC2c expression*			
High *versus* low	1.98	1.94–2.26	0.02
			
*MiR-101 expression*			
High *versus* low	1.60	1.14–2.23	0.02
