# Supplementary material for: CASC2c as an unfavorable prognosis factor interacts with miR-101 to mediate astrocytoma tumorigenesis
Source: Cell Death Dis. 2017 Mar 2;8(3):e2639–. doi: 10.1038/cddis.2017.11 (PMC5386525; doi:10.1038/cddis.2017.11)
Supplement: Supplementary Information [file cddis201711x1.docx]

**CASC2c as an Unfavorable Prognosis Factor Interacts with miR-101 to Mediate Astrocytoma Tumorigenesis**

Changhong Liu^1, 2^, Yingnan Sun^1,2^, Xiaoling She^3^, Chaofeng Tu^2^, Xiping Cheng^4^, Lin Wang^5^, Zhibin Yu^2^, Peiyao Li^2^, Qing Liu^6^, Honghui Yang^7^, Guiyuan Li^1, 2^, Minghua Wu^2*^

1. Hunan Provincial Tumor Hospital and the Affiliated Tumor Hospital of XiangyaMedical School, Central South University, Changsha 410013, Hunan, China

2. Cancer Research Institute, School of Basic Medical Science, Central SouthUniversity; Key Laboratory of Carcinogenesis and Cancer Invasion, Ministry of Education; Key Laboratory of Carcinogenesis, Ministry of Health. Changsha410078, Hunan, China

3. Second Xiangya Hospital, Central South University, Changsha 410013, Hunan,China

4. Regeneron Pharmaceuticals, Tarrytown, NY 10591, USA

5. Department of Neurosurgery, University of Michigan Medical School, Ann Arbor, MI 48109, USA

6. Xiangya Hospital, Central South University, Changsha 410013, Hunan, China

7. Third Xiangya Hospital, Central South University, Changsha 410013, Hunan, China

* To whom correspondence should be addressed: Minghua Wu, Cancer ResearchInstitute, School of Basic Medical Science, Central South University, Changsha, Hunan 410078, Tel: 86-731-82355401. Fax: 86-731-82355401. E-mail: wuminghua554@aliyun.com


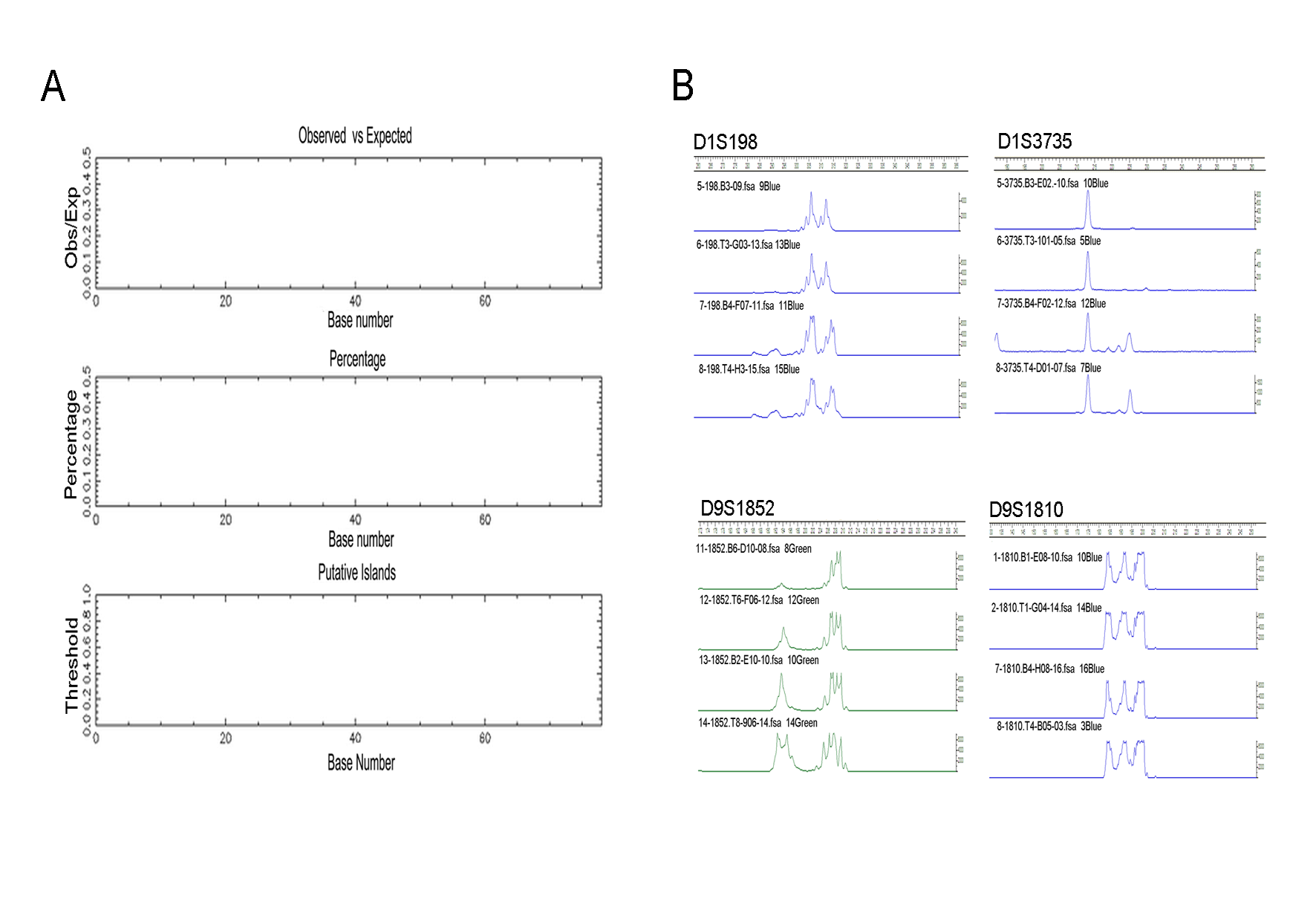


**Supplementary Figure 1.No CpG island and no loss of heterozygosity were found in pre-miR-101.**

A No CpG island of pre-miR-101 was predicted by EMBOSS (European Molecular Biology Open Soft Suite).

B No loss of heterozygosity was found in chromosome 1p31 (pre-miR-101-1) and 9p24 (pre-miR-101-2) by touchdown PCR in U251 cells.

**
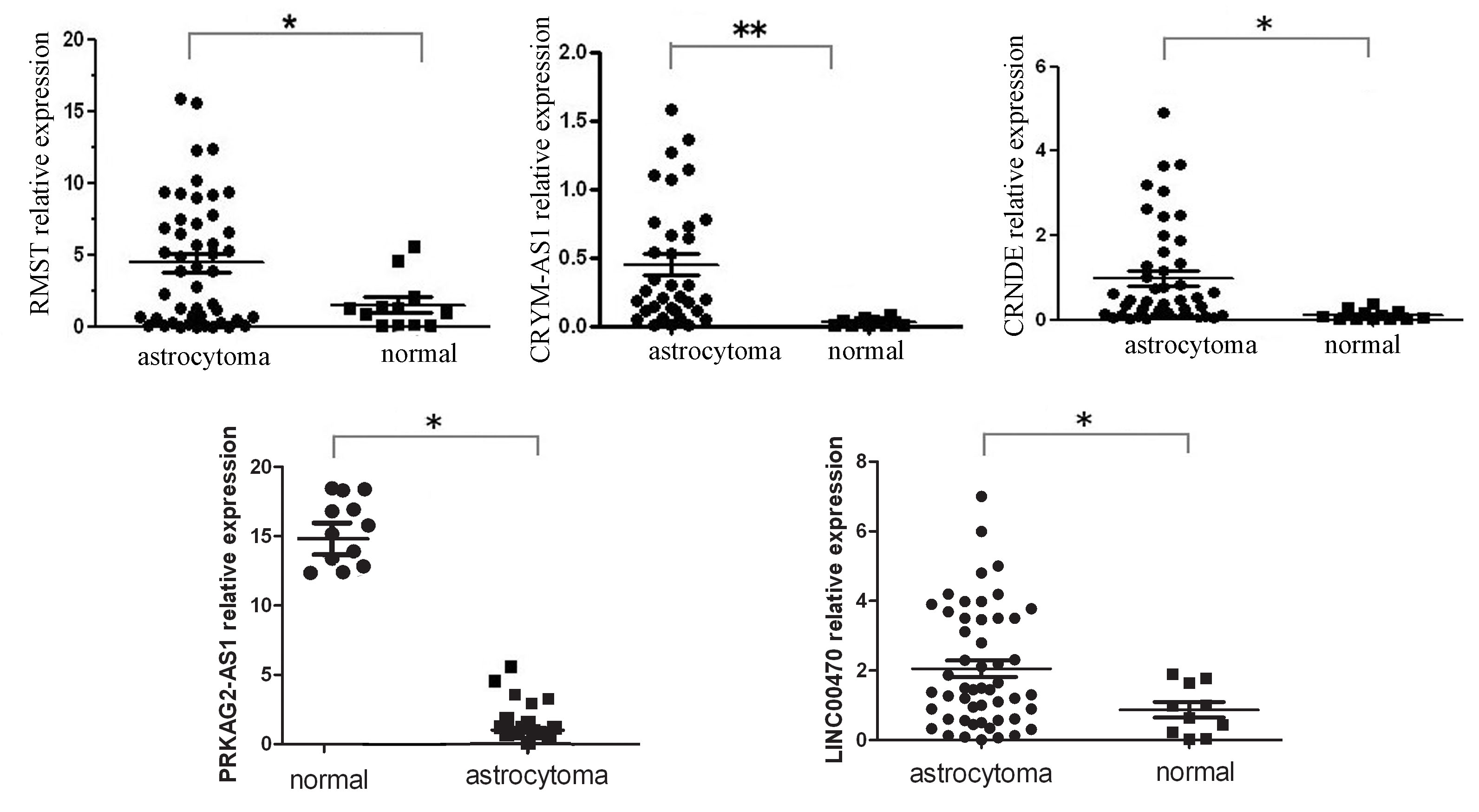
**

**Supplementary Figure 2**.**Relative expression levels of LncRNAs .**

The expression of RMST, CRYM-AS1, CRNDE, PRKAG2-AS1, LINC00470 in astrocytoma and normal brain were detected by real time qPCR. Data shown are the mean ±SEM of three independent experiments;**P*< 0.05, ***P*< 0.01.


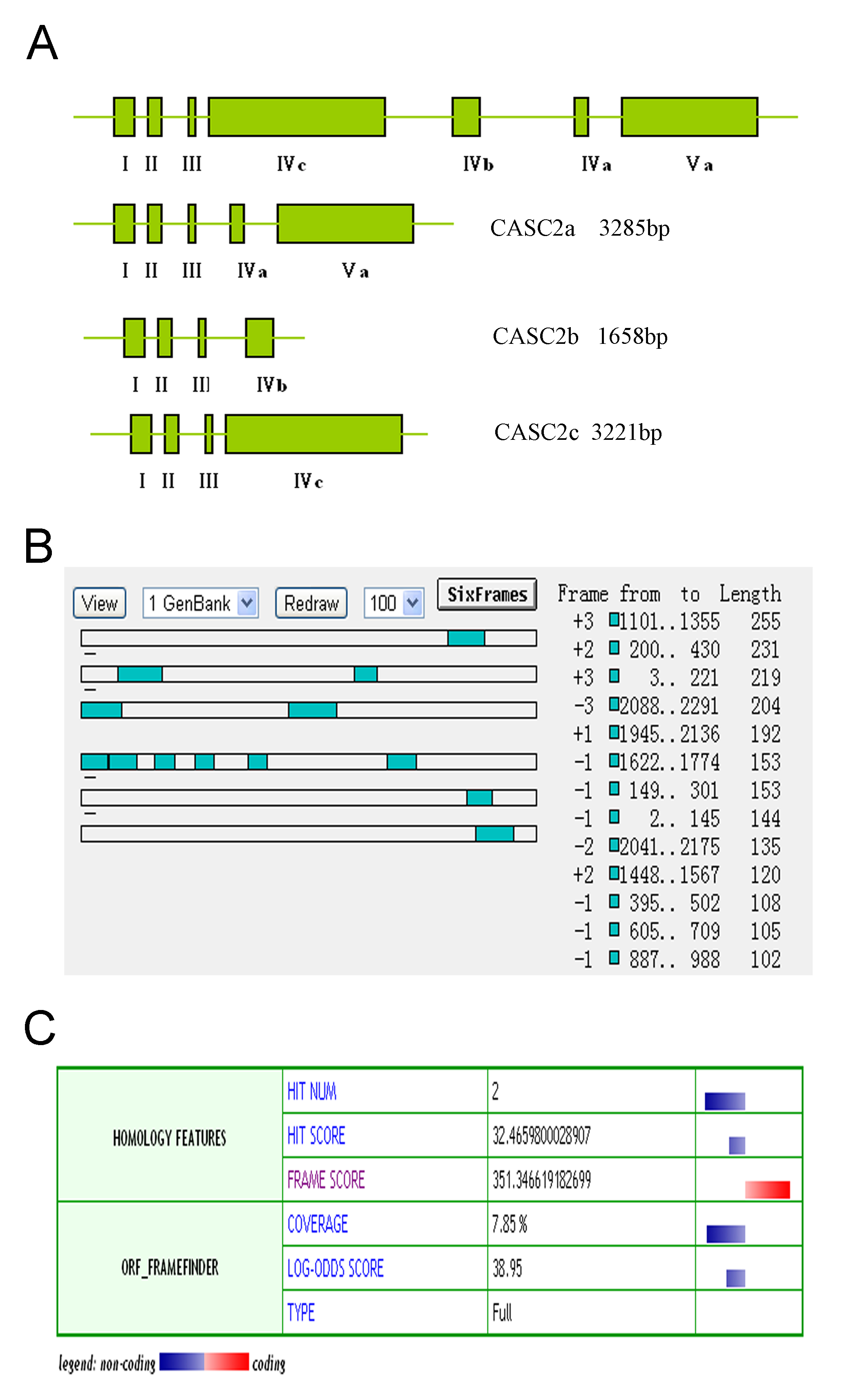


**Supplementary Figure3. The analysis of CASC2 gene family and CASC2c protein domains by online software.**

A CASC2 gene family was analyzed by genomic and cDNA sequence comparisons.

B CASC2c encoded amino acids analysis through ORF finder software in online.

C No known protein domains were predicted by Coding Potential Calculator Software.

**
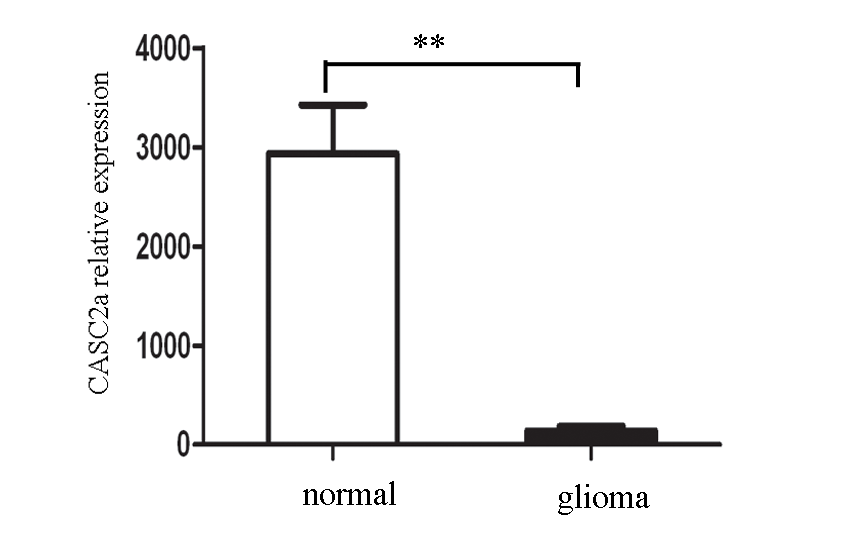
**

**Supplementary Figure 4 .Relative expression levels of CASC2a.**

The expression of CASC2a in glioma and normal brain was detected by real time qPCR. Data shown are the mean ±SEM of three independent experiments;*** *P* < 0.001.


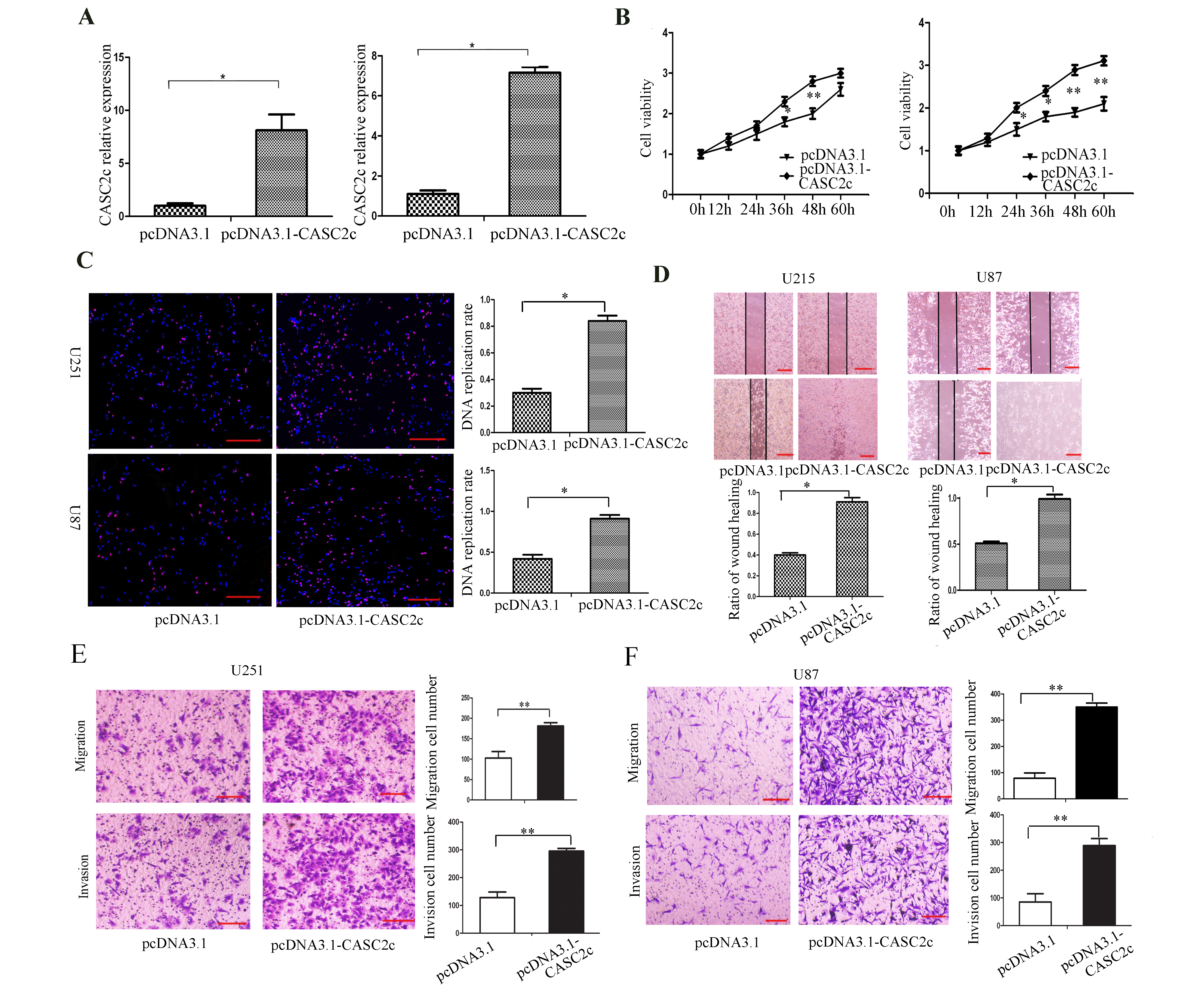


**Supplementary Figure 5 .CASC2c overexpression promotes the malignant characteristic of astrocytoma cells.**

A CASC2c expression levels were evaluated using real time qPCR in pcDNA3.1/CASC2c-transfected U251 and U87 cells. Data shown are the mean ±SEM of three independent experiments;**P*< 0.05.

B CCK-8 assaywas performed to determine the viability of U251 and U87 cells overexpressing CASC2c.Data shown are the mean ±SEM of three independent experiments; **P*< 0.05, ** *P* < 0.01.

C EDU assay was applied to assess cell proliferation of U251 and U87 cells overexpressing CASC2c.Data shown are the mean ±SEM of three independent experiments; red scale bars, 200μm; **P* < 0.05.

D Wound healing assay measured cell migration of U251and U87 cellsoverexpressing CASC2c.Data shown are the mean ±SEM of three independent experiments; red scale bars, 200μm; ***P*< 0.05.

E and F Transwell assay and matrigel-coated transwell assay were performed in U251 and U87 cells overexpressing CASC2c. Data shown are the mean ±SEM of three independent experiments; red scale bars, 50μm; ** *P*< 0.01.


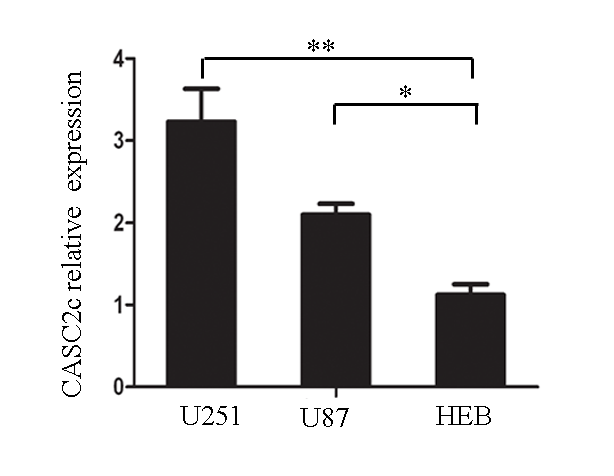


**Supplementary Figure 6.Relative expression levels of CASC2c.**

CASC2c expression levels were evaluated using real time qPCR in U251,U87 and HEB cells.

Data shown are the mean ±SEM of three independent experiments;**P*< 0.05, ** *P*< 0.01 .

**
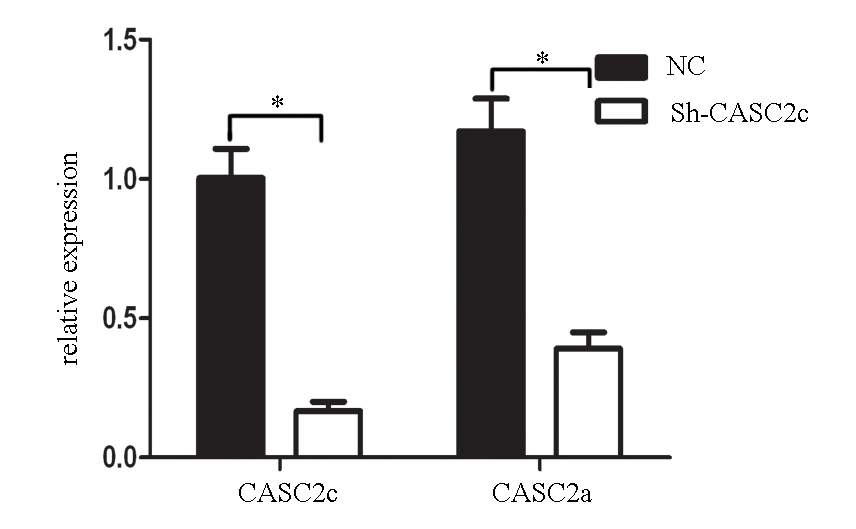
**

**Supplementary Figure 7.Relative expression levels of CASC2c and CASC2a.**

CASC2a and CASC2c expression levels were evaluated using real time qPCR in control/sh-CASC2c-transfected U251 cells. Data shown are the mean ±SEM of three independent experiments ;**P* < 0.05.


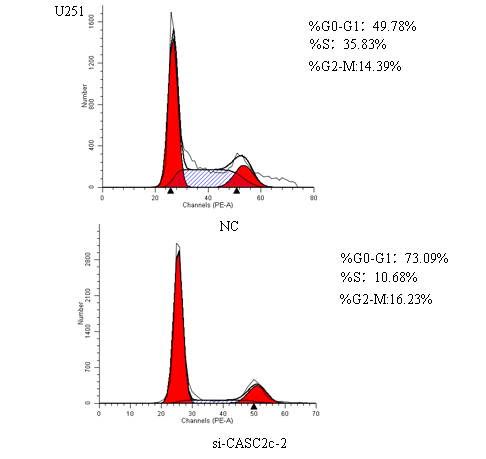

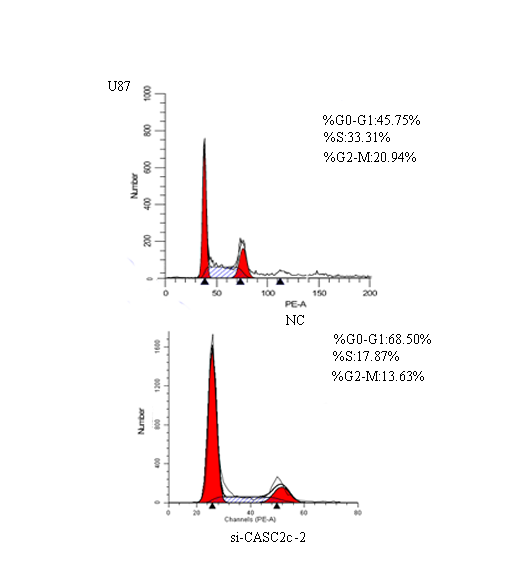

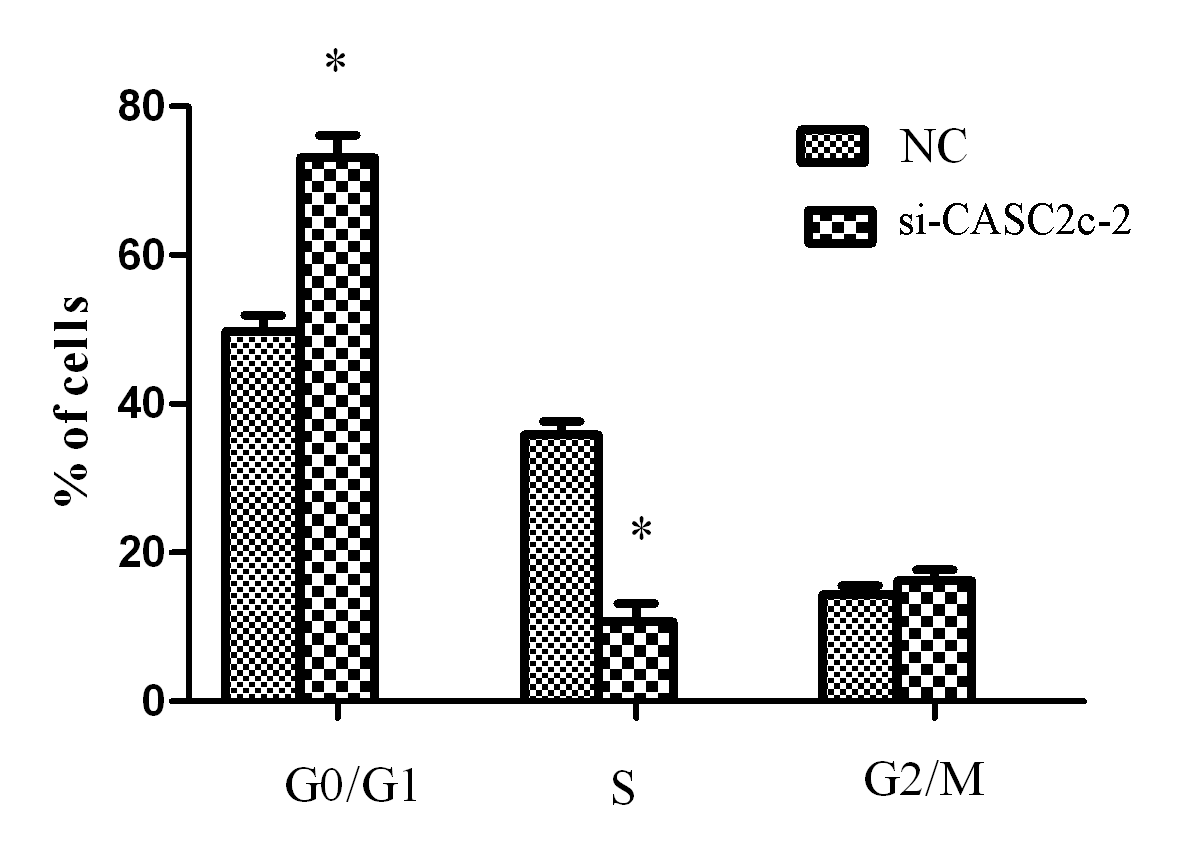

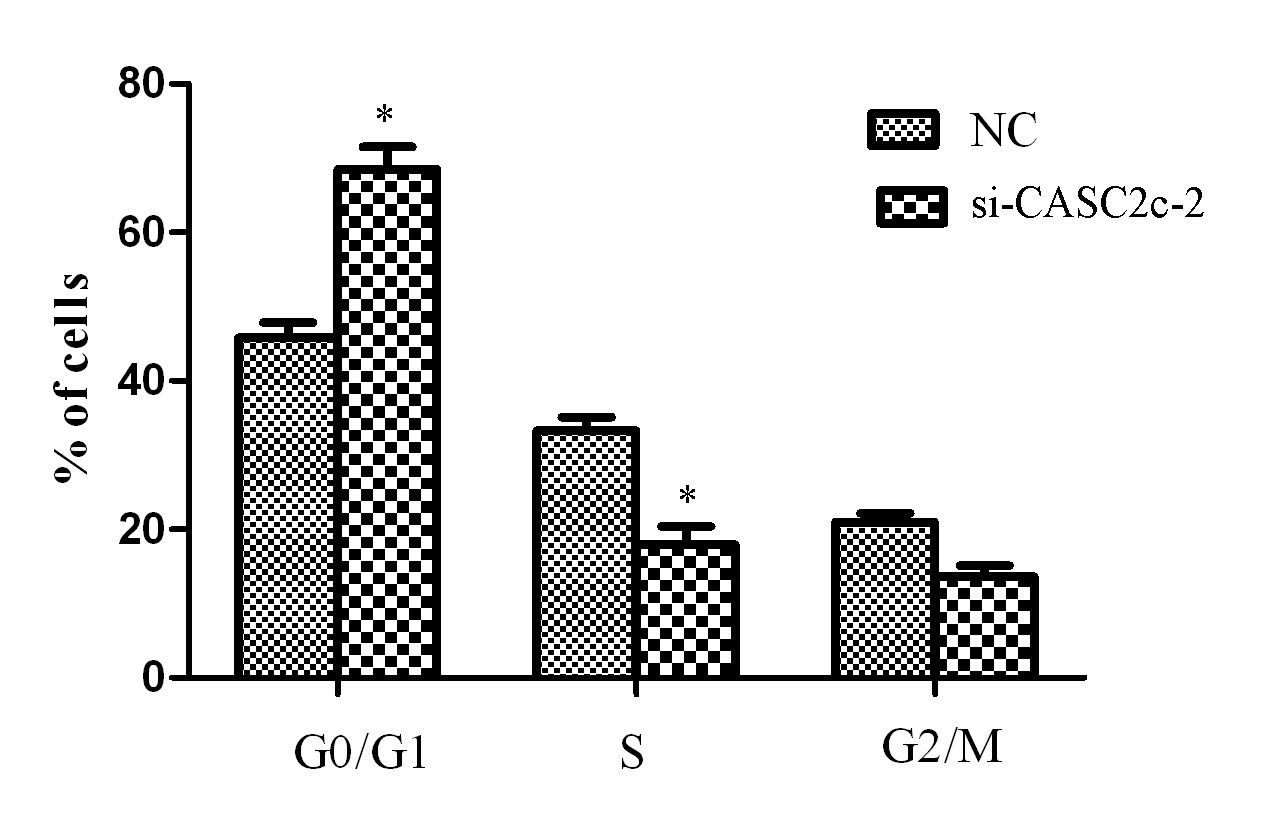


**Supplementary Figure 8. Effect of si-CASC2c-2 on cell cycle of U251 and U87 cells.**

Effect of si-CASC2c-2 on cell cycle of U251 and U87 cells were detected by flow cytometry .Data shown are the mean ±SEM of three independent experiments;**P*<0.05.

**
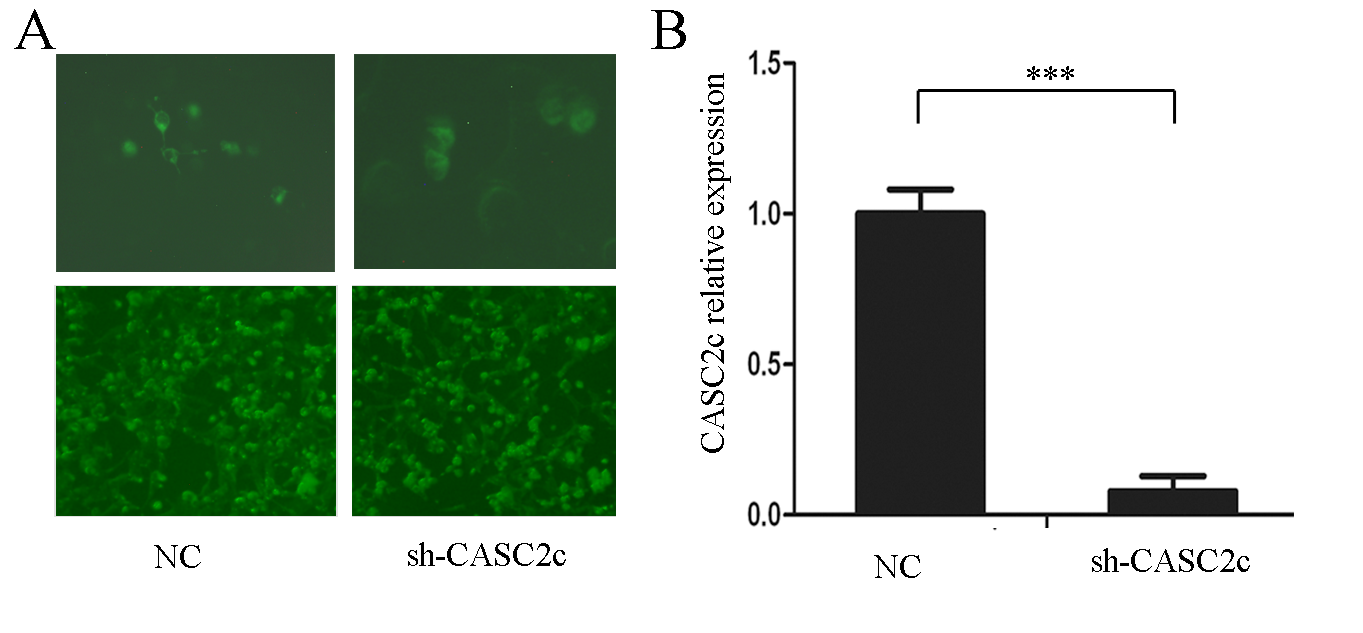
**

**Supplementary Figure 9. U251 cells with stablely expressed-sh-CASC2c were contructed.**

A U251cells were transfected with NC and sh-CASC2c, the positive clones were screened by G418.

B The expressive levels of CASC2c in positive cells were detected by real time qPCR.Data shown are the mean ±SEM of three independent experiments;*** *P* < 0.001.

**
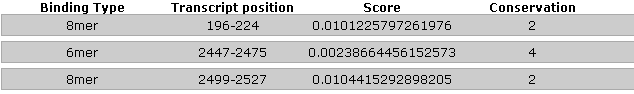
**

**Supplementary Figure 10 .Three binding sites exist between CASC2c and miR-101.**

**
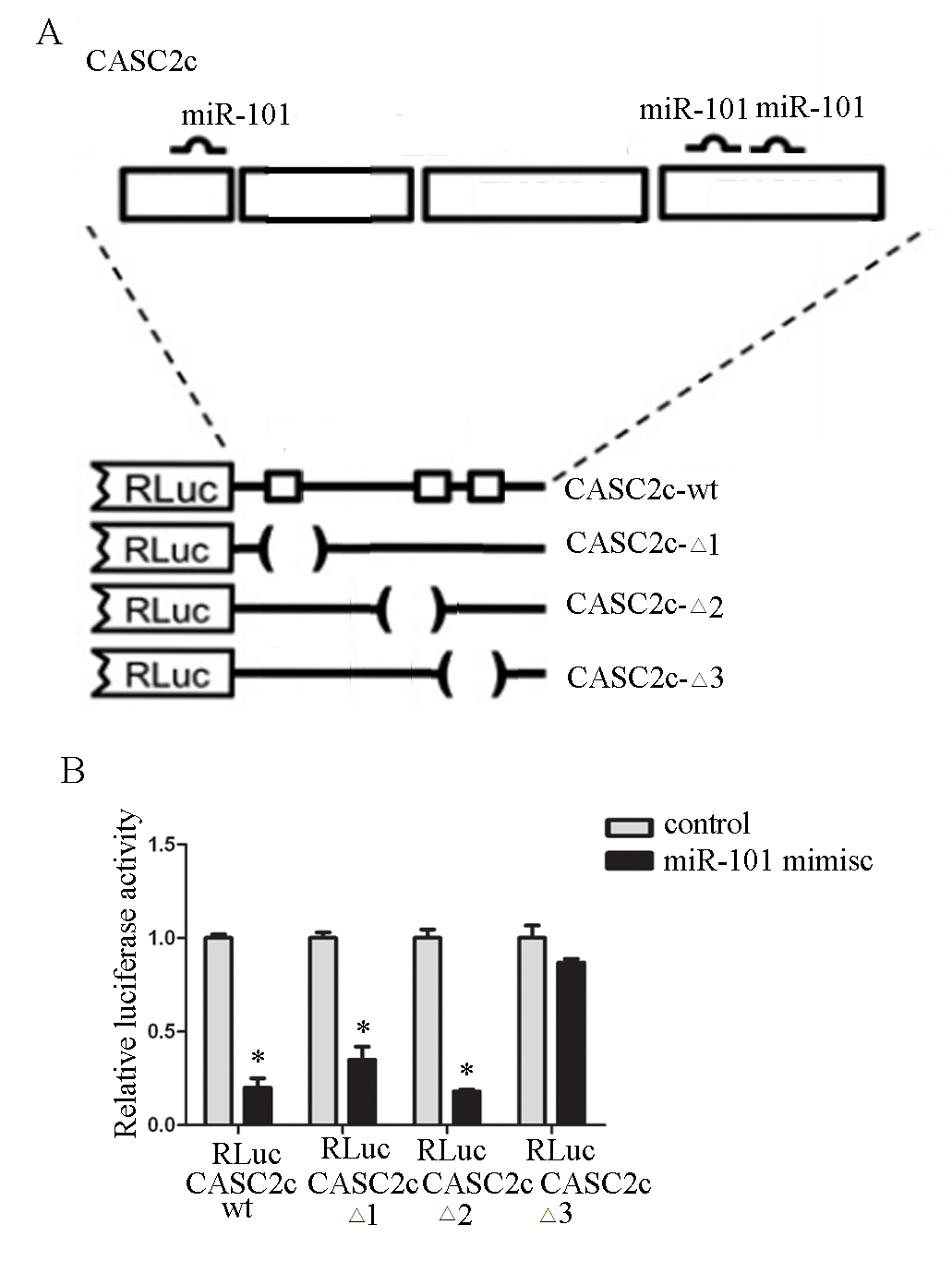
**

**Supplementary Figure 11.The binding between miR-101 and CASC2c.**

A Upper, Positions of miR-101 binding sites on CASC2c.Below, CASC2c(RLuc-CASC2c-wt) and mutant derivatives devoid of miR-101 binding sites (RLuc-CASC2c-△1，RLuc-CASC2c-△2 and RLuc-CASC2c-△3 ) .

B The values of luciferase measured after transfection plasmids. Data shown are the mean ±SEM of three independent experiments;**P*<0.05.

**
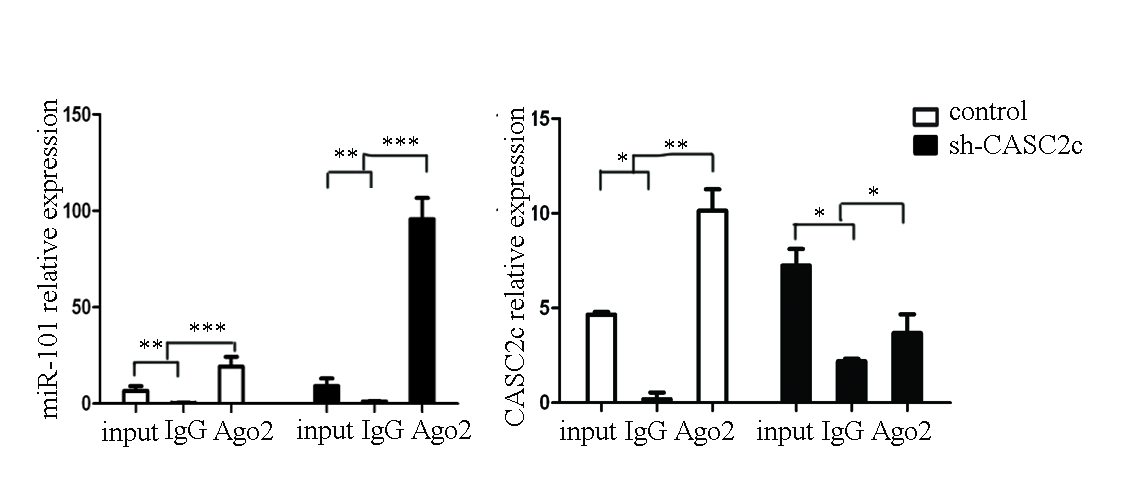
**

**Supplementary Figure 12. Relative expression levels of miR-101 and CASC2c.**

The expression of CASC2c and miR-101 were measured by real time qPCR.Data shown are the mean ±SEM of three independent experiments;**P*<0.05,** *P* < 0.01,*** *P* < 0.001.

**
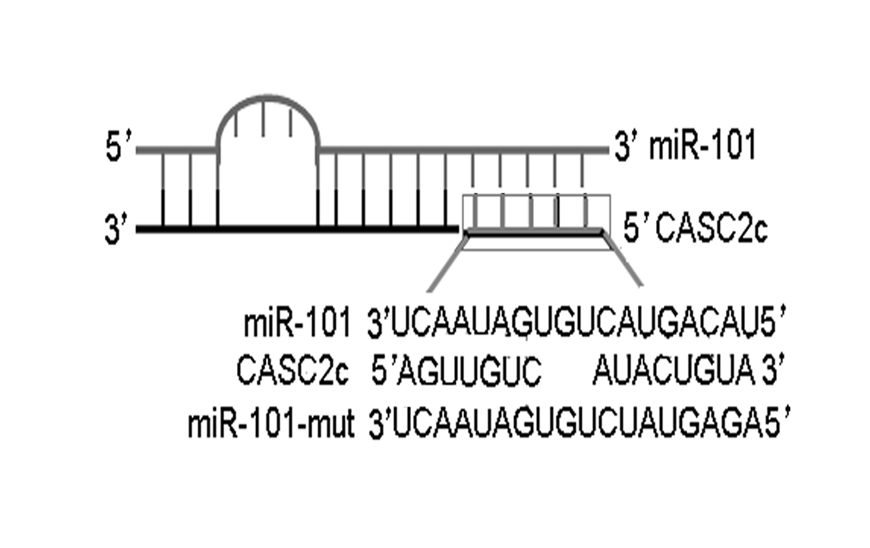
**

**Supplementary Figure 13. Alignment of CASC2c base pairing with miR-101.**

**
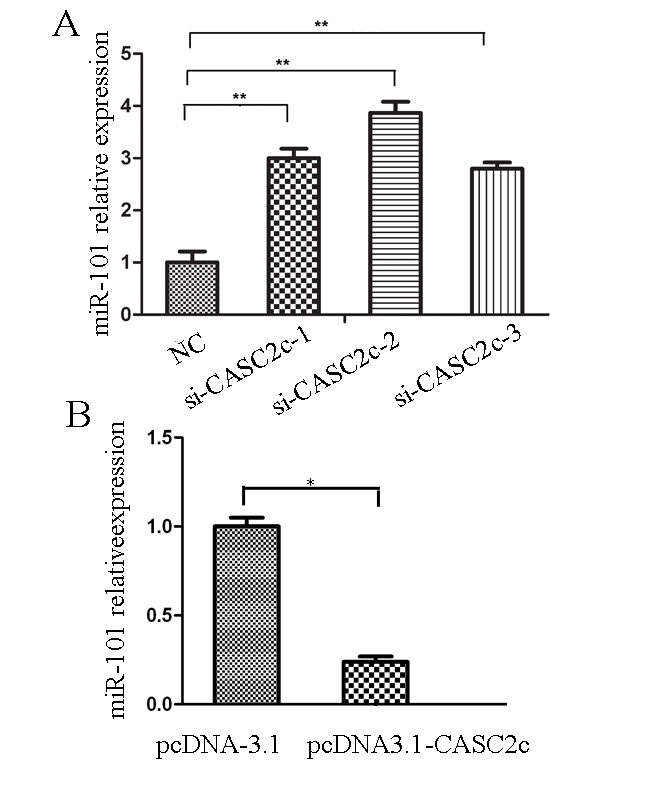
**

**Supplementary Figure 14.Relative expression levels of miR-101 .**

A miR-101 expression level was evaluated using real time qPCR in control/si-CASC2c-transfected U251 cells. Data shown are the mean ±SEM of three independent experiments; ***P*<0.01.

B miR-101 expression level was evaluated using real time qPCR in pcDNA3.1/ pcDNA3.1-CASC2c-transfected U251 cells.Data shown are the mean ±SEM of three independent experiments;**P*<0.05.

**
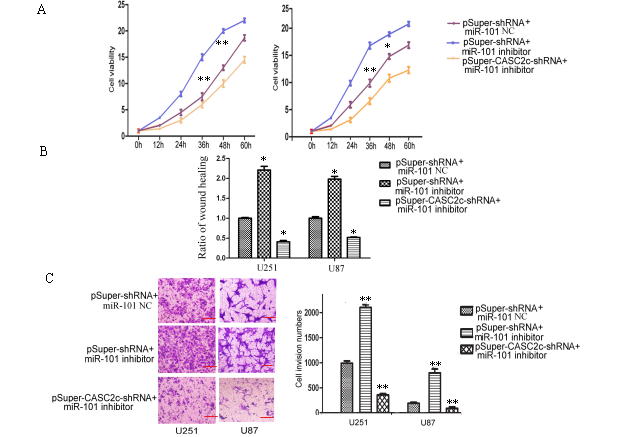
**

**Supplementary Figure 15.Downexpression of CASC2c largely reversed miR-101-induced inhibitory effects on astrocytoma cells.**

A Downexpression of CASC2c partly reversed miR-101-induced inhibition of proliferation in U251 and U87 cells determined by CCK-8 assay.Data shown are the mean ±SEM of three independent experiments; **P*<0.05, ***P*<0.01.

B Downexpression of CAS2c largely reversed miR-101-induced suppression of migration and in U251 and U87 cells detected by wound healing.Data shown are the mean ±SEM of three independent experiments; **P*<0.05

C Downexpression of CASC2c partly reversed miR-101-induced inhibition of invasion in U251 and U87 cells detected by transwell assay.Data shown are the mean ±SEM of three independent experiments; red scale bars,50μm;***P*<0.01.


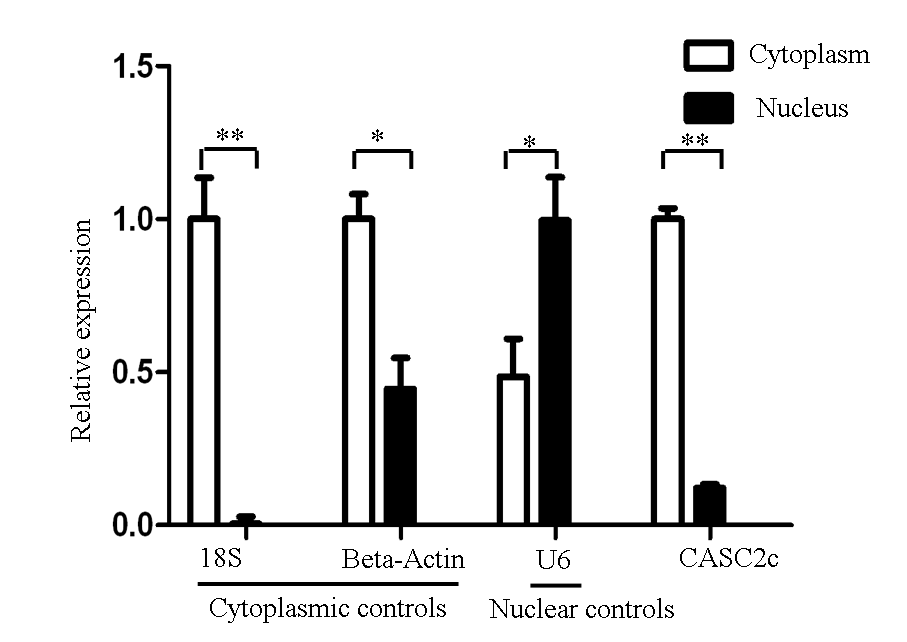


**Supplementary Figure 16. Subcellular localization of CASC2c.**

Relative expression of CASC2c was evaluated using real time qPCR, cytoplasmic control RNAs 18S, β-Actin, and nuclear control RNAU6 isolated from nuclear and cytoplasmic cell fractionations of U251 cells.Data shown are the mean ±SEM of three independent experiments; **P*<0.05,***P*<0.01.
