# Supplementary material for: CASC2c as an unfavorable prognosis factor interacts with miR-101 to mediate astrocytoma tumorigenesis
Source: Cell Death Dis. 2017 Mar 2;8(3):e2639–. doi: 10.1038/cddis.2017.11 (PMC5386525; doi:10.1038/cddis.2017.11)
Supplement: Supplementary Table 1 [file cddis201711x2.docx]

**Supplemental Table 1.The primers used in this study**

| **Gene name** | **Forward / Reverse primer(5’- 3’)** |
| --- | --- |
| CASC2c | F:5’-TGGGTATTAGCCGACAGT-3’  R:5’-CTCCGTTGGTTATTGAAAGT-3’ |
| pri-miR-101 | F:5’GAGTAATGCAGCCACCAGAA-3’  R:5’GCCATCCTTCAGTTATCACAGTA-3’ |
| pre-miR-101 | F:5’-GCCCTGGCTCAGTTATC-3’  R:5’-GCCATCCTTCAGTTATCACAGTA-3’ |
| CPEB1 | F:5’-TGGGTATTAGCCGACAGT-3’  R:5’-CTCCGTTGGTTATTGAAAGT-3’ |
| Dicer | F:5’-GGTGGTCCACGAGTCACAAT-3’  R:5’-TAGCACTGGCTTCGTTTCGT- 3’ |
| GAPDH | F:5’-ATCAAGATCATTGCTCCTCCTGAG -3’  R:5’- CTGCTTGCTGATCCACATCTG -3’ |
