# Supplementary material for: CASC2c as an unfavorable prognosis factor interacts with miR-101 to mediate astrocytoma tumorigenesis
Source: Cell Death Dis. 2017 Mar 2;8(3):e2639–. doi: 10.1038/cddis.2017.11 (PMC5386525; doi:10.1038/cddis.2017.11)
Supplement: Supplementary Table 2 [file cddis201711x3.docx]

**Supplemental Table 2.LncRNAs that bind with miR-101 were predicted by DIANA LAB and miRanda**

| **Number** | **Name of LncRNA** | **miTG score** |
| --- | --- | --- |
| 1 | XIST | 0.852 |
| 2 | GCFC1-AS1 | 0.796 |
| 3 | STARD4-AS1 | 0.785 |
| 4 | LINC00230A (FAM224A) | 0.767 |
| 5 | NEAT1 | 0.716 |
| 6 | RMST | 0.648 |
| 7 | CRNDE | 0.638 |
| 8 | CASC2 | 0.634 |
| 9 | CRYM-AS1 | 0.630 |
| 10 | PRKAG2-AS1 | 0.628 |
| 11 | MIR4500HG | 0.627 |
| 12 | LINC00309 | 0.620 |
| 13 | LIFR-AS1 | 0.600 |
| 14 | LINC00657 | 0.912 |
| 15 | LINC00271 | 0.868 |
| 16 | POU4F1-AS1 | 0.811 |
| 17 | PWRN1 | 0.741 |
| 18 | LINC00470 | 0.624 |
