# Supplementary material for: CASC2c as an unfavorable prognosis factor interacts with miR-101 to mediate astrocytoma tumorigenesis
Source: Cell Death Dis. 2017 Mar 2;8(3):e2639–. doi: 10.1038/cddis.2017.11 (PMC5386525; doi:10.1038/cddis.2017.11)
Supplement: Supplementary Table 3 [file cddis201711x4.doc]

**Supplemental Table 3. Expression of CASC2c and miR-101 between astrocytoma and normal tissures**

|  |  | CASC2c |  |  | miR-101 |  |  |
| --- | --- | --- | --- | --- | --- | --- | --- |
| Variable | Cases | low | high | P | low | high | P |
| Tumors | 80 | 18(22.5%) | 62(77.5%) |  | 66(82.5%) | 14(17.5%) |  |
| Normal tissures | 18 | 11(61.1%) | 7(38.9%) | 0.015 | 5(27.8%) | 13(72.2%) | 0.001 |
